# Biogeopolitics of COVID‐19: Asylum‐Related Migrants at the European Union Borderlands

**DOI:** 10.1111/tesg.12448

**Published:** 2020-07-01

**Authors:** Jussi S. Jauhiainen

**Affiliations:** ^1^ Department of Geography and Geology University of Turku Turku FI‐20014 Finland; ^2^ Institute of Ecology and the Earth Sciences University of Tartu Tartu Estonia

**Keywords:** Biogeopolitics, COVID‐19, asylum, migration, Greece, Turkey

## Abstract

In biogeopolitics, the key state stakeholders develop and aim to accomplish their geopolitical goals by (mis)management and biopolitical governance of vulnerable population. In this paper, this population refers to asylum‐related migrants who use or aim to use an asylum request as their entry mechanism to the European Union. This paper explores the emergence of biogeopolitics at the EU borderland between Turkey and Greece during the COVID‐19 pandemic in 2020. Statistics about irregular migration from Turkey to Greece, field observations in Lesvos (Greece) as well as media and social media discussions about COVID‐19 in Lesvos are analysed. In the biogeopolitics of COVID‐19, the governance and (mis)management of asylum‐related migrants include policies and practices to let these migrants to live or die, including regulating illegal border‐crossings, everyday living conditions at the reception centres, and actions regarding the pandemic. The COVID‐19 pandemic was used as an additional tool to foster biogeopolitics.

## Graphical abstract

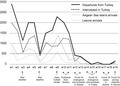

## Introduction

The COVID‐19 pandemic has geopolitical dimensions. The top priorities dictating the actions against the spread and the outcomes of the COVID‐19 pandemic are focused on states, state territories, state borders and citizens within a given state. These actions fostered the role of states as the key geopolitical references to imagine, discuss and act upon the world. Furthermore, the media and the related statistical information portrayed and discussed this pandemic in a way that highlights the division of the world into states.

These sovereign geopolitical territorial units were not able to prevent the arrival of the virus (with the propagandistic exceptions of North Korea and Turkmenistan) due to border‐crossings. Therefore, among the first actions taken by the governments were ‘lockdowns’. Most countries prevented people from moving and migrating across its borders and imposed strong regulations concerning how people can get together in the public space of that country. Particularly strong concerns were expressed over the pandemic’s deathly impact on individual countries’ demography (i.e. mortality rates and numbers among citizens), as well as its devastating impact on the economy of individual countries (i.e. unemployment, bankruptcies and rising public social welfare burden), resulting from the rapid downturn in the global economy. Much of the observations were thus about each state, as well as its sovereignty and measures to protect citizens.

In such a state‐centric discussion about COVID‐19, much less attention has been paid to displaced people. Displaced people, in this context, are tens of millions of people who had to leave their homes to seek economic, social and political security and/or asylum in another country. To ask for asylum, one must leave one’s country of origin, cross the border to another country and request asylum there or travel further to do it in another country. These displaced people flee one state and expect protection from another state (United Nations 1951). However, they are in between two countries, still on the move or in the asylum process, having limited and only temporary formal protection by any state.

People in flight (irregular migrants, asylum seekers and other similar groups) are vulnerable, often seen as ‘aliens’. Irregularity of migration is defined by the states: irregular migrants are those who enter or remain in the country without the legal right and consent of the authorities. When serious global and national challenges hit a country, such as an economic recession or the COVID‐19 pandemic, displaced people’s welfare is not prioritised like the welfare of the proper citizens of that country. During this pandemic, asylum seekers and displaced people before they request asylum were not neglected entirely; controversially, their vulnerability and lack of protection are actively utilised by selected key state stakeholders for other purposes to legitimate the state geopolitical interests.

This paper discusses biogeopolitics of COVID‐19. In the context of the paper, biogeopolitics refers to the key state stakeholders’ aims and practices to accomplish their preferred geopolitical goals by biopolitical governance and (mis)management of asylum‐related migrants (people using or aiming to use asylum request as the entry mechanism to the European Union). The movement of these migrant bodies belongs to the broader geopolitical order in the territories with which these bodies are acquainted and the political, social and biological function of these bodies (i.e. the biopolitics of asylum‐related migration). The empirical geographical setting for this paper is the southeastern borderland of the EU, namely the border areas between Greece and Turkey, particularly the Aegean Sea islands, including Lesvos. The COVID‐19 pandemic emerged as an unexpected factor, but it soon became part of broader biogeopolitics to foster the goals of specific geopolitical interests in the EU borderlands.

The focus of the paper is on irregular asylum‐related migration from Turkey to Greece (January–May 2020), the governance of asylum‐related migrants and their agency during the early stage of the COVID‐19 pandemic. The paper thus answers the following research questions: (i) How did irregular migration from Turkey to Greece develop during the early stages of the COVID‐19 pandemic?; (ii) How the asylum‐related migrants were governed in the Reception and Identification Centre of Moria in the Greek island of Lesvos in the wake of the COVID‐19 pandemic?; and (iii) What kind of agency did the asylum‐related migrants in Moria show, if any, related to the emergence of COVID‐19? The asylum‐related migrants’ mobility, activities and agencies are analysed within the framework of biogeopolitics connected to broader state‐oriented geopolitics at the EU borderlands and the impact and use of COVID‐19 on it.

The empirical material for the paper was gathered and analysed using mixed methods. The data include: statistical information about irregular migration between Turkey and Greece from January–May 2020 (departures of irregular migrants from the Turkish coast, their interceptions by the Turkish border guards and police before their entering the EU, and the arrivals of asylum‐related migrants to the Greek Aegean Sea islands as well as state‐led mobilisations of these migrants for such border‐crossings); field observations (supported by a survey and interviews with asylum seekers) related to their governance in the Reception and Identification Centre of Moria (a major gateway for asylum‐related migrants to the EU) in November 2019; and various media and social media material regarding the COVID‐19‐related activities of asylum‐related migrants (especially in Moria) in March–April 2020.

## Background

During the 2010s, the southeastern EU borderlands became a very geopolitically sensitive area. This related to the ongoing serious political challenges and military actions in the nearby territories of Syria and Turkey, and even up to the border of Iran, to name a few. One result of such challenges was a large number (up to 800,000) of asylum‐related migrants arriving through Turkey to Greece in 2015 (Eurostat 2017). Eventually, the EU and Turkey released a joint EU‐Turkey statement on 18 March 2016. It aimed to establish a cooperative relationship between the EU and Turkey and to compel Turkey to cease irregular migration via Turkey to Europe. The EU promised to compensate Turkey with around 6 billion euro to enhance the situations of such migrants as must now remain in Turkey. Many of them were from Syria, Afghanistan and sub‐Saharan nations. According to the statement, among other things, Turkey agreed to prevent irregular migration from its territory to the EU, and especially to the Greek Aegean Sea islands. Turkey also agreed to intercept irregular migrants in Turkish waters and take them all back, and facilitate the rapid return of those crossing from Turkey into Greece and not in need of international protection (European Commission 2016; European Council 2016). Essentially, this was about top‐down management of migration, categorising the populations according to their countries of origin, and preventing the non‐Europeans from entering the EU. This is about the governance of migration and utilising asylum‐related migrants in biogeopolitical approaches.

In 2016, the number of arrivals from Turkey to Greece declined rapidly, up to 90 per cent compared with the previous year. However, these migrants needed to find other routes to the EU. In fact, in 2017, the Central Mediterranean route from Libya to Italy became the most frequented. After Italy implemented strong preventative measures against asylum‐related migration, the most frequented route in 2018 was the Western Mediterranean route from Morocco to Spain. Then in 2019, the Eastern Mediterranean route from Turkey to Greece became the most frequented one again (UNHCR 2019b; 2020).

Another measure to regulate the asylum‐related migration was establish the EU migration ‘hotspots’ located in the migrants’ first arrival places such as the island of Lesvos in Greece at 10 km from the Turkish coast and the island of Lampedusa in Italy at 280 km from the Libyan coast. The ‘hotspot approach’ was launched in 2015 in the EU to manage exceptional numbers of asylum‐related migrants arriving in the EU (European Commission 2017). Accordingly, the initial asylum processes, such as identification of individuals, first hearings and later transfer decisions needed to be conducted at the EU hotspots on the border, such as in Lesvos and Lampedusa (Alpes *et al*. 2017). However, the asylum inspection procedures were slow, requiring individual migrants to remain in these hotspot reception centres from months to more than one year. The facilities in these centres were often poor, rendering the everyday lives of asylum seekers challenging. Such (un)intended prolongation of the asylum processes at the EU migration hotspots means a kind of ‘pop‐up governance’, coined by Papada *et al*. (2019). This includes disorganised management of migration with abruptly introduced practice‐based mechanisms. The perceived crisis, its specific events and temporary emergency are governed with flexible filling of temporal and spatial gaps in the governance capacity.

The migration regulation and the hotspot approach are part of broader biogeopolitics – connecting the real physical bodies of asylum‐related migrants and the body of the state. The key state stakeholders aim and practice to accomplish their preferred geopolitical goals and order by biopolitical governance and (mis)management of asylum‐related migrants (i.e. the people in flight using or aiming to use asylum request as the entry mechanism to the EU).

In connection with this, the aforementioned EU‐Turkey statement of 18 March 2016 is an example of the expansion of EU asylum and migration regulation and policies outside of the territory of the EU, in this case to Turkey. At the heart of such policies is the governance of asylum‐related migration and migrants: a desire to decide which kinds of people may enter the EU, who is allowed to remain inside the EU, and how. One action is to prevent their arrival as such by limiting irregular border‐crossings to the EU, in this case from Turkey. Another action is the surveillance and securitisation of EU external borders with combined military‐humanitarian interventions in the surveillance, rescue and containment of the movement of asylum‐related migrant populations in the Aegean Sea (Tazzioli 2016). This include several EU‐wide past, current and future programmes and activities by Frontex (i.e. Mare Nostrum and Triton) and the European Border and Coast Guard Agency in the Mediterranean that govern the maritime border areas between Greece and Turkey. This also includes the modes by which irregular migrants are rescued and by whom. A further action concerns how the asylum seekers (i.e. people who managed to cross the EU border and request asylum in the EU) are (mis)managed during the asylum process, which may lead to a residence permit in the EU but more often ban of entry to the EU and consequent removal of the former asylum seeker from the EU territory. Though emergence of the COVID‐19 pandemic was unexpected, it was soon utilised in biogeopolitics, as evidenced in this paper.

Biopolitics is one operational tool in biogeopolitics. In the mid‐1970s, Foucault (2003) explained in the biological modernity that the aim of the state is to govern biological processes and the life of human beings as a species. Often in research, biopolitics has been used in rather binary modes, in which strict top‐down practices are implemented over these migrants. Migrants have remained without (much) agency in these situations. The most critical scholarship elaborates on philosopher Giorgio Agamben’s concepts. There, the biopolitically organised legal system decides the extent to which human rights apply to asylum‐related migrants in their ‘bare lives’, reduced to minimum of survival (Agamben 1998; Ek 2006). In this process, asylum‐related migrants are disqualified from their earlier existence, numbered and reclassified, and finally translated into a biopolitical mass without individuals (see Aradau & Tazzioli 2019). In the separation of politically conditioned and biological lives, the migrants’ bodies are ‘Othered’ from the forms of life of the proper citizens. The migrants’ bodies become conditioned instruments subjected to various restrictions and obligations. The authorities use these – often racialised (Anderson *et al*. 2019) –bodies for specific purposes making them residues of the state (see Agamben 2015).

Ultimately, biopolitics of the asylum‐related migrants is about keeping these alien asylum populations alive or letting them die (see Foucault 2003) in the dangerous circumstances they face during the asylum‐related journeys. Such contradictory ‘anthropogenetic’ project (Agamben 2015), on the one hand, includes remaining passive in their rescue activities in the sea (or even driving over their vessels as reported by the media, NGOs, migrants, etc.), thereby letting people drown. In the unhealthy conditions during the journeys, including being obliged to remain in overcrowded reception centres (or informal camps), asylum‐related migrants easily become ill. Failing to take proper care of their illnesses result in deaths, also from violence among these migrants in these nerve‐breaking circumstances. COVID‐19 was another challenge for asylum‐related migrants. They had to meet and travel with groups of people in Turkey before joining together for the dangerous passage to Greece. Furthermore, they had to remain in very densely populated reception centres along their journeys, both inside and outside the EU. On the other hand, rescuing, safeguarding and feeding these migrants is based on their ‘Othering’ and not letting these migrants to share the same space with the EU citizens. In the end, the state keeps these migrants alive but not in liveable conditions. The authorities’ and migrants’ (re)action on COVID‐19 is another example of this keeping alive/ letting die dilemma.

In the (mis)management of asylum‐related migrants, their basic biological needs are taken care of, but the aim is to leave them without political agency. However, these migrants as human beings cannot be fully deprived of their agency. Some of them self‐organise and create actions of resistance, show solidarity among themselves and support the new political identities that grow from their seemingly hopeless circumstances (Martin *et al*. 2019). Ultimately, asylum‐related migrants have goals in their lives, and for example reaching the Turkish coast or arriving at a Greek island are steps toward these goals. Furthermore, even small self‐made and self‐managed items and activities (such as erecting a tent, making food outside of it, or providing small everyday services to fellow migrants) are expressions of their mundane agency and hope for a better future. Furthermore, the top‐down migration management is unable to cease completely the irregular migration to the EU. These migrants had already demonstrated agency by being able to alter their trajectories according to the possibilities of crossing the national borders and those of the EU.

However, the COVID‐19 pandemic was an unexpected factor influencing the regulation of migration and the (mis)management of these migrants, as discussed in the empirical section. On the one hand, it was not considered possible to expose purposefully the migrants in Turkey or the Greek islands to SARS‐CoV‐2 virus and leave them into such ‘bare life’ (see Agamben 1998; Ek 2006). On the other hand, these potentially infected migrants may be used as an instrument to foster the achievement of specific geopolitical goals in the southeastern EU borderlands. This paper thus highlights the impact of COVID‐19 on the numbers and migration patterns of irregular migrants, the governance practices regarding these migrants and the migrants’ agency.

## Data and Methods

The empirical material for this paper was gathered and analysed using mixed methods. The first material consists of statistical data about irregular migration between Turkey and Greece from January–May 2020. Acquiring such data and verifying their reliability requires cross‐checking many sources. The Greek border authorities release daily information about the arrival of irregular migrants (most of whom ask for asylum) to the Greek islands in the Aegean Sea (see National Coordination Centre 2020). In addition, the NGO Aegean Boat Rescue provides weekly data that also includes the number of intercepted people and boats at sea before their arrival to the Greek waters and the numbers of migrants in these islands (Aegean Boat Report 2020). It also provides the number of arrived people and boats. In addition, there is information from the NGO Mare Liberum, which observes the irregular migration and rescue activities between Turkey and Lesvos. From crosschecking these reliable sources are traced the departures of irregular migrants from the Turkish coast, their interceptions in the Turkish waters, and their arrivals to the EU (i.e. to the Greek Aegean Sea islands). This data from January to May 2020 was analysed with longitudinal descriptive statistical methods comparing the migration numbers with the main events regarding the relationships between the EU, Greece and Turkey, as well as the development of the COVID‐19 pandemic in Greece and Turkey. The migration‐related material includes also recent media discussion (including fake news) about the prospect that infected migrants would be pushed from Turkey to the EU to spread COVID‐19 (Nedos 2020).

The next empirical material consists of field observations about the management and governance of asylum seekers in Lesvos, Greece. These observations were supported by the results of a survey of 625 asylum seekers there, as well as related thematic interviews with about 40 of them, conducted both by the author and two research assistants in November 2019 (i.e. before the evidence of COVID‐19 in Lesvos). The survey and interviews were about the everyday lives of asylum seekers, their journey to Lesvos and migration aspirations forward as well as their Internet and social media uses. However, the general results from the survey and interviews fall out of the scope of this paper. In this paper, the particular focus is on the Reception and Identification Centre of Moria (RICM). This material provides insights into the everyday life situations and the biopolitical governance of about 20,000 asylum‐related migrants in the RICM. It is globally notorious because of its challenges of being very overcrowded, having poor sanitation and health conditions and being a site fraught with insecurity and violence for the everyday lives of many migrants. The purpose is to contextualise the situation in the event of the arrival of COVID‐19 to Lesvos and the context in which the exposure to COVID‐19 may be fatal for many migrants in the RICM. The main methods for the field observation notes was theory‐driven content analysis backed with empirical results of survey and interviews (see Jauhiainen & Vorobeva 2020).

The third set of material consists of various recent media, NGO and the asylum seeker social media and other types of posting regarding COVID‐19 and asylum‐related migrants in Greece and Turkey. As discussed in the empirical results, this material illustrates the top‐down and bottom‐up practices in the prevention of COVID‐19 in the RICM. Furthermore, the material shows how asylum seekers’ agency and new political identities emerged with the potential threats of COVID‐19, however, sometimes remaining symbolic without any guarantee of material security. The media and social media texts were analysed with theory‐driven content analysis.

## Results

The early stages of the global COVID‐19 pandemic in 2020 accentuated major geopolitical tensions between Turkey and the EU, particularly between Turkey and Greece. The pandemic was a surprise, but the states took it into their arsenal for their geopolitical battles and utilised for it the asylum‐related migrants at the EU southeastern borderland. The two issues discussed here are: first, the impact of the COVID‐19 pandemic on irregular migration and the use of asylum‐related migrants to threaten to disperse COVID‐19 to other countries; second, the (mis)management and biopolitical governance of asylum‐related migrants in the RICM and top‐down and bottom‐up actions against COVID‐19 there.

### Regulating irregular migration between Turkey and Greece during the COVID‐19 pandemic

The geographical location of the Greek Aegean Sea islands close to the Turkish coast is attractive for asylum‐related migrants to access the EU. The islands were the main gateways to the EU in 2015 and again in 2019 as the most popular route to cross the Mediterranean (UNHCR 2020). However, during the COVID‐19 global outbreak in early 2020, asylum‐related migration changed. There was the usual seasonal fluctuation in migration mostly due to weather‐related reasons. COVID‐19 had an impact on migration and migrants and there were also attempts to use it in irregular migration to create threats to achieve geopolitical goals.

**Figure 1:**
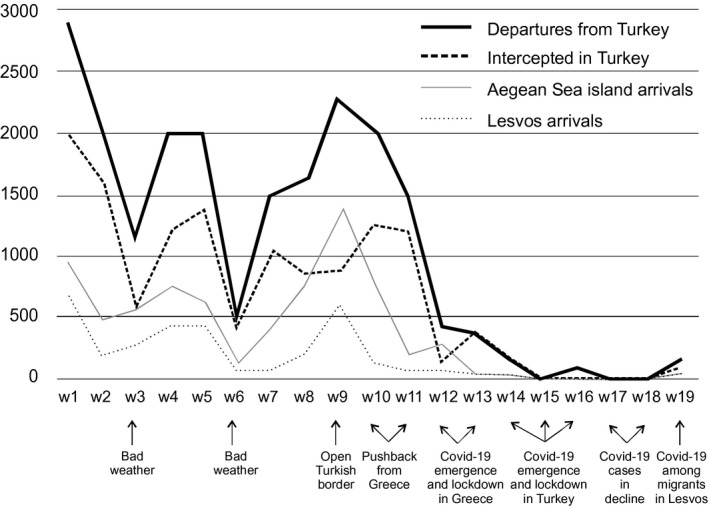
Irregular migration from Turkey to the Aegean Sea islands of Greece in January–May 2020. *Source*: Modified from Aegean Boat Report (2020) and National Coordination Centre (2020).

Regarding seasonal changes in irregular migration, fewer asylum seekers arrive on the Greek Aegean Sea islands in winter than during the rest of the year. During winter, many days, mainly heavy winds and sometimes excessive rain make it too risky to accomplish the required few‐hours sea passage with densely packed dinghies from the Turkish coast to the islands. In addition, for Afghans (who are among the largest groups to migrate to the islands), it is very challenging to illegally cross the Iranian–Turkish border mountains when it is very cold, and there is a lot of snow. In January–February 2020, irregular migration was slightly higher compared with the year before (Figure 1). On the average, 79 persons arrived daily to the Greek islands (including 34 persons to Lesvos). Sometimes there were no arrivals for several days (Aegean Boat Report 2020; National Coordination Centre 2020), again, mostly because of the bad weather.

However, the COVID‐19 pandemic came to play a significant role in irregular migration from Turkey to Greece. During early 2020, when the first cases of COVID‐19 were already present in China but awareness was almost non‐existent in the EU (including Greece) and Turkey, the geopolitical tensions between Turkey and the EU intensified. In particular, they were connected to the ongoing war in Syria, the activities of Turkey in this war, and the position of the EU regarding the war and the Turkish military interventions. The Turkish military had occupied areas of the Syrian territory and created buffer zones close to Turkey. Many Syrians had to escape from Idlib to these areas, and some also continued to Turkey that increased concerns in Turkey. Furthermore, intolerance towards Syrians in Turkey grew, creating discontent with the leading political party of Turkey, AK (‘Justice and Development’), with which the president of Turkey, Recep Tayyip Erdoğan, was affiliated. From 2014 onward, Syrians had gained temporary protection status in Turkey. Part of the EU‐Turkey statement deal was that the EU would compensate Turkey to host these Syrians (European Council 2016). However, over the years, their number grew to 3.6 million, and they could not return to Syria with the exception of rather non‐voluntary migration to the areas occupied by Turkey (Deutsche Welle 2018: McKernan 2019). The initially large EU reimbursement – up to 6 billion euro – came to mean a monthly assistance of a few tens of euro per one Syrian in Turkey.

Turkey needed political support for its presence in Syria and wished that many Syrians would leave its territory. Syrians in Turkey became a tool to put pressure on the EU. In fact, from the beginning, several scholars criticised the EU‐Turkey statement as a deal that the EU made in 2016 in an unfavourable political context, fearing continuous immigration by millions of asylum seekers. Turkey did regulate irregular migration, so the EU became hesitant to openly criticise Turkey, including when in the aftermath of the so‐called coup d’état in 2016, Turkish authorities did not respect human rights when punishing individuals suspected of treason and when the Turkish military invaded Syria in 2016. Instead of sustainable and effective policies to handle asylum‐related and labour‐related migration, the EU created a mechanism to hold a certain number of asylum‐related migrants in Turkey (Haferlach & Kurban 2018). The performance and performance indicators mattered only partially in the operationalisation of the statement (Tantardini & Tolay 2020). For example, the practice of immediate return of asylum seekers from the EU to Turkey, mentioned in the statement, never worked (at least officially). In 2016–2019, only about 2,000 asylum seekers were returned to Turkey to transfer them to their countries of origin (UNHCR 2019a). However, Turkish authorities and some international NGOs claimed that the Greek border authorities had informally pushed immediately back a few tens of thousands of asylum‐related migrants without giving them the opportunity to ask for asylum (Christides & Lüdke 2019). As discussed later in this paper, in March–April 2020, the Greek border authorities openly practised such immediate pushbacks in contravention of the principle of non‐refoulement in international law and the European Convention of Human Rights.

In the attempts to increase the geopolitical power of Turkey, President Erdoğan and the Turkish government expected support from countries and international bodies (including the EU) to legitimate Turkey’s territorial expansion to the Syrian territory and the pushback of Kurdish people and militants from the Turkish border areas. However, while the Turkish presence in these territories was tolerated, there was no uniform international political support for this occupation. The occupation was clearly against international laws and agreements, and unanimous international support for an occupation of foreign territory would have set an inconvenient precedent for similar actions by other countries in other territories. Irritated with such a position and due to Turkish domestic politics, President Erdoğan had several times threatened the EU to withdraw from fulfilling the 2016 EU‐Turkey statement and let millions of migrants travel from Turkey to Greece. The president and the leading political party were losing popularity due to the continuing presence of Syrians in Turkey while the country was facing economic challenges.

On 28 February 2020, a major attack against the Turkish forces in Syria took place, leading into tens of casualties of Turkish soldiers. Infuriated, President Erdoğan and the Turkish government launched several military attacks against the military forces that were supporting the Syrian government. With the impact of Russia, a ceasefire was soon established. However, President Erdoğan pushed the geopolitical pressure on the EU further. On 29 February he pronounced ‘We opened the door’, that is, that the Turkish borders were open to the EU (Stevis‐Gridness & Gall 2020) without agreeing on this with Greece or the EU. Transport was organised for migrants to reach the land border with Greece and the western coast. Within a few days, thousands of migrants (many of whom were Afghans, Syrians, sub‐Saharan Africans and Pakistanis) gathered together. They were encouraged to pass the Turkish border guards to reach the actual borderline between Greece and Turkey. Similarly, migrants were encouraged to immediately take boats towards the Greek Aegean Sea islands, including Lesvos. Asylum‐related migrants were intentionally used to reach geopolitical goals of Turkey in the wider Mediterranean and the Middle East.

At the land border with Greece violent clashes and pushbacks took place and only a few hundred migrants managed to break through to Greece (BBC 2020b). However, at sea the situation was different though pushbacks took place as well. Following the rapid dispersal of the news that the Turkish border was open to the EU, in two days only (on 1–2 March), 1,514 asylum‐related migrants reached the Aegean Sea islands, including 612 Lesvos. It was a tenfold growth to the situation in January–February (Figure 1). During the nine days (21–29 February) before this event, 567 migrants reached the Greek islands, and of them, 184 reached Lesvos. However, during the nine days following the event (3–11 March), only 209 migrants reached the Greek islands, and of them, 42 migrants (one boat only) reached Lesvos (National Coordination Centre 2020). In fact, the Greek border authorities managed to prevent much of the entrance attempts of asylum‐related migrants. The authorities even conducted violent immediate pushbacks at land and sea borders that were globally witnessed in the broadcast news (BBC 2020b). Several NGOs claimed that the Greek police and border authorities continued pushbacks also during the COVID‐19 pandemic in March–April, even from reception centres and pre‐removal detention centres (Border Monitoring Violence Network *et al*. 2020). Another measure was the decision of the Greek government to temporarily suspend the possibility to ask for asylum in Greece. This was clearly against international and EU asylum legislation, and several organisations presented their criticism against such decisions, including those from the EU (European Council 2020). Some international crews belonging to Frontex did not agree to push back the migrants (Tritschler 2020). However, the hard practice yielded an immediate result. The number of arrivals dropped as quickly as they had risen (Figure 1). In addition, those migrants who arrived at the Greek islands in March were taken directly under surveillance and shipped to mainland Greece without opening their asylum process. They were placed in dedicated centres to wait for their removal from Greece.

The processes discussed above took place just before the outbreak of COVID‐19 in Greece and Turkey. Before 12 March, fewer than 100 people had been identified to be infected with the SARS‐CoV‐2 virus in Greece, none of them had died, and there were no cases yet in Turkey (Worldometers 2020). When it became evident that the migrants could not break through to Greece, Turkey silently withdrew from its attempt to encourage migrants to cross the border to the EU. The Turkish border authorities returned to their usual semi‐efficient interception of irregular migrant border crossings (see Figure 1). The lockdown in Turkey since 18 March and several restrictions on intra‐regional transport since 28 March created constraints also on irregular migration. However, COVID‐19 soon came to play an important role in the irregular migration and it was used to have a geopolitical impact at the EU borderlands.

In March 2020, about 4,400 irregular migrants left the Turkish coast towards the Greek islands. The Turkish border guards and the police intercepted about 3,000 of them before they crossed the maritime border, so about 1,400 migrants managed to reach the islands. However, in mid‐April, for the first time since 2015, not even one irregular migrant reached the Greek islands during one week (Figure 1). In addition, during that week, the Turkish authorities intercepted at sea only 30 migrants who attempted to leave Turkey irregularly to go to these islands (Aegean Boat Report 2020). COVID‐19 had reached Turkey. In fact, by the end of March, the identified COVID‐19 cases were over 10,000 in Turkey, and the number of casualties attributed to COVID‐19 in Turkey surpassed 1,000 on 10 April, and the identified cases passed 100,000 on 23 April (Worldometers 2020).

The global and European significance of the COVID‐19 pandemic became evident by mid‐March to the highest political elites of Turkey. President Erdoğan should have met face‐to‐face on 17 March with German Chancellor Merkel, French President Macron and the British Prime Minister Johnson to discuss the future of the EU‐Turkey statement, needed to be renewed rather soon. However, they met only virtually because of the COVID‐19 pandemic (France 24 2020). The attention of the European leaders had shifted from the EU borderlands to the pandemic that did not go unnoticed by the Turkish president though Turkey had then less than 100 identified COVID‐19 cases (Worldometrics 2020).

While the expanding COVID‐19 posed a major challenge to Turkey, it also presented an opportunity to utilise asylum‐related migrants in an attempt to threaten the EU and lessen the internal political pressures. Namely, Syrians numbered over 3.5 million in Turkey, and there were a few hundred thousand irregular migrants from other nations. On the one hand, these immigrants would be a potential threat to the Turkish population because many of them moved and gathered in groups, thus potentially being exposed and exposing the virus. On the other hand, infected irregular migrants could bring COVID‐19 from Turkey to the Greek islands (and the EU), with tens of thousands of asylum seekers. Such a possibility of utilising displaced people for geopolitical goals also involved Syrians arriving recently from the war‐torn Syria. Due to continuous fighting and bombing in the Idlib area, more than a million Syrians had to escape from there in 2019, early 2020 towards Turkey because other areas near‐by were under the control of the Government of Syria and its military. These people were kept in large semi‐official camps close to the Turkish border (Ahmad 2020). The COVID‐19 pandemic also reached Syria, and the first verified case was found there on 22 March – however, there might have been cases earlier (Worldometers 2020). Turkey had closed the border to Syria already on 11 March.

Afghans were another large group of immigrants (more than 200,000) in Turkey. However, most of them were irregular migrants because they could not get asylum in Turkey due to restrictions in Turkey’s agreement with the Convention Relating to the Status of Refugees (United Nations 1951). The arrival of irregular Afghans to Turkey diminished in 2020 because the border between Turkey and Iran was more strictly controlled and closed on 23 February due to the emergency of COVID‐19 in Iran. Furthermore, to limit the spread of COVID‐19, the Iranian authorities reduced the possibilities for intra‐Iran travelling and controlled the main travel routes between Iranian regions. Such impediments to travel to the Turkish border also affected Afghans from both Afghanistan and Iran.

In mid‐April 2020, news spread through various media and social media (including rumours and fake news) that the Turkish authorities had gathered hundreds of Syrians from their provisional settlements and camps in the southern borderlands of Turkey and brought them to the western coast of Turkey, to the sites from which the sea passages start (Nedos 2020). Facilitating the travel of these migrants to the EU would require cooperation between public and private authorities as well as illegal actors. As usual, smugglers could organise the few‐hours sea passage in dinghies that take about 30–45 passengers. Each boat that arrived full of passengers would generate an income of 20,000–40,000 euro for smugglers, as the price of the sea passage was about 500–1,000 euro (Jauhiainen & Vorobeva 2020). Such purposeful sending of infected asylum‐related migrants from Turkey to the Greek islands would create a humanitarian catastrophe in the conditions of vulnerable asylum seekers in overcrowded reception centres on the Greek islands, as discussed in the section below. However, from early April to early May, the attempts to reach the Aegean Sea islands irregularly from Turkey were exceptionally few (Figure 1). After 1 April, the first boat with asylum‐related migrants arrived at Greek islands on 6 May (National Coordination Centre 2020).

### Biopolitics and agencies at the Reception and Identification Centre of Moria facing COVID‐19

The Reception and Identification Centre of Moria (RICM) on the island of Lesvos is globally among the most notorious reception centres for asylum seekers. It is run by the Greek national authorities, and the UNHCR is also significantly involved in the actual management. Over the past several years, it has been frequently present in the media, most often due to challenges in the everyday lives of the asylum seekers there. In 2015, over half a million asylum‐related migrants travelled from Turkey through Lesvos to farther into the EU. By 2020, the total number of such migrants who had travelled through Lesvos was close to one million, which is a large number for an island with 90,000 people.

During the first half of 2019, the number of arrived migrants and those transferred to mainland Greece was in balance. However, in the autumn of 2019, the arrivals grew quickly, but the transfers did not, so the RICM became very overcrowded. In early 2020, it held about 20,000 people whereas its facilities had been designed for fewer than 3,000 people. The site inside the walls (a former military garrison having mostly containers for 8–12 asylum seekers) had been packed for some years already. However, the extension area outside the walls (where the asylum seekers live throughout the year in open air in tents for 4–10 people) grew significantly during 2019: from 2,000 asylum seekers in January to over 15,000 in December (Aegean Boat Report 2020; National Coordination Centre 2020).

Part of the biopolitics regarding asylum‐seekers is the slow asylum process and remaining stuck in the RICM in which their opportunities for smooth everyday lives are limited. Such challenges start from the EU migration hotspot approach (European Commission 2017). First, after the asylum‐related migrants cross the maritime border to the Greek waters, they are spotted and rescued either at sea by accredited NGOs or international or Greek border guards or at the coast by accredited NGOs. Then they are brought to the RICM to be registered as asylum seekers (if they ask for asylum, as practically all migrants do). Their information (including their fingerprints) is inserted in the EURODAC system indicating that they asked for asylum in Greece. This is to prevent the same person’s application being processed in another EU member state.

After the registration, the person is assigned a place in the RICM. If there is not a free place inside the centre, then a place in a tent in the immediate surrounding area outside the centre is indicated and the asylum seeker is (usually) provided with a blanket. The RICM has about one toilet per 100 persons, one shower per 120 persons and one medical doctor per 10,000 persons. Rubbish is spread around the area, and there is a shortage of clean water. The population density in the RICM (covering the areas both inside and outside the walls) is over 10,000 people per square kilometre. In early 2020, Afghans were the largest ethnic group (78%), followed by Syrians (8%) and Somalis (4%), and about 42% of asylum seekers were children (for a general description of the RICM, see Jauhiainen & Vorobeva 2020).

In the next stage of the asylum process, the asylum seeker waits until the first asylum interview is conducted with him or her by the asylum authorities – this waiting usually takes a few months. From day to day, he or she receives a couple of bottles of water per day, as well as daily meals (if there is no shortage of them) for which he or she has to queue for 1–2 hours inside the RICM. Each asylum seeker also receives a monthly allowance (around 80–90 euro) after a few months in the RICM. After the first asylum interview, the asylum seeker waits for transfer to mainland Greece and further administrative practices in the asylum process. The transfer takes place usually after a few months following the interview. Most asylum seekers thus spend several months and even more than a year in the RICM. Meaningless waiting for an undetermined time creates multiple forms of social harm, and according to Iliadou (2019), time and waiting are forms of continuing organised, legitimised and routinised everyday violence as a state tool of control and deterrence posed upon asylum seekers. Those with vulnerabilities or small children may have a chance to be removed to other and better reception centres in Lesvos. However, there is a long queue for this removal as well.

In such (mis)management, the asylum seekers in the RICM have to live in continuous emergency situations. The authorities’ practices tend to deny a promising future for asylum‐related migrants (Ramadan 2013; Anderson *et al*. 2019), and in fact, only a minority of them will ever get residency in the EU. These migrants are portrayed to live on the margins of human life as passive and voiceless recipients of assistance, lacking a home, nation and citizenship, as well as proper agency, voice and face (Turner 2016). However, such a top‐down perspective does not grasp that reception centres for asylum‐related migrants can be sites of agency, resistance, solidarity and new political identity (Martin *et al*. 2019).

The global outbreak of the COVID‐19 pandemic rapidly created a serious threat to the lives of asylum seekers in the RICM as well as in other densely populated reception centres in Greece. However, in the RICM they live on an island, so access to the island and the mobility of the inhabitants and asylum seekers could be more easily managed. By the immobilisation of people, the Greek state could do much to prevent the spread of COVID‐19 to the RICM and likewise by the mobilisation of irregular migrants to cross the border, the Turkish state could increase the threat of such spreading. Soon after the first COVID‐19 case was identified in Lesvos (among the inhabitants and not the asylum seekers), on 9 March, several international organisations and the media, as well as by some NGOs present in the RICM recognised the potential threat of COVID‐19 to the asylum seekers. A pressure was created to take up measures against it and there were even calls to evacuate the RICM as well as other overcrowded reception centres (Médecins sans Frontières 2020). A month later, on 19 April, Greek authorities launched a plan to move on up to 2,380 elderly and ailing asylum seekers from the Aegean Sea islands to the mainland to reduce the risk of a virus outbreak (InfoMigrants 2020) – another practical approach of flexible pop‐up governance (see Papada *et al*. 2019) to react abruptly on the perceived crisis. Still after a few weeks, the plan had not yet been realised showing the lack of efficient strategy and management.

However, the national and local authorities started several cautionary measures. In early March an obligatory 14‐day quarantine was imposed on the island people potentially infected and those arriving from abroad (including members of various international NGOs operating in the RICM). Access to the RICM by those other than asylum seekers living in the RICM or the permanent staff working there was limited on 19 March. The NGOs had only a restricted access – for good and for bad – to the asylum seekers. In Lesvos and its main centre, Mytilene, restaurants, cafes and other places for public gathering, even churches, were closed. The air and ship connection from the mainland Greece to island was limited, and later a national lockdown was implemented on 23 March. In May it became obligatory to wear masks in public places. The first COVID‐19‐related casualty in Lesvos took place on 29 March, again among the inhabitants (Corona 24 News 2020).

The asylum‐related migrants did not remain passive in waiting for the potential arrival of the pandemic to the RICM. The NGOs and the authorities started to inform asylum seekers in the RICM about the virus and the simple means (such as washing hands and wearing a face mask) to prevent catching it with informative posters in various languages. Some water tanks were put in place to help in the basic hygiene. However, also the asylum seekers showed an immediate agency, thus being able to escape from their top‐down categorisation into a uniform mass without agency. By 18 March, the asylum seekers had set up a mask factory and awareness teams inside the RICM. They produced face masks in small self‐made sweatshops (Fallon 2020a). These masks of cotton would not totally protect against getting COVID‐19. Furthermore, they were not enough for the entire migrant population being more than 20,000 on the island. Some asylum seekers made short critical films about their everyday challenges, and some of these films gained international coverage, for example, through the BBC (BBC 2020c). This was an expression of their new political agency in the threatening circumstances of the pandemic.

The first identified cases of COVID‐19 inside the asylum seeker reception centres in Greece occurred on 1 April. However, until early May, no cases were identified in the RICM. The arrivals of people outside of Greece to Lesvos were substantially limited during the national lockdown and quarantine was implemented. In the three weeks before mid‐April, only one boat with asylum‐related migrants arrived at Lesvos, the smallest number in several years, and from 2 April to 5 May no migrants arrived. By early May, the virus had been (temporarily) erased from the island. A major threat remained with the potential arrival of infected asylum‐related migrants from the near‐by Turkey. By 12 May in Turkey, there were 1,700 confirmed cases per one million inhabitants (Worldometers 2020), however, no public information about the situation among the more than four million refugees and irregular migrants there.

In March–April, during the arrival of the COVID‐19 pandemic, the authorities, NGOs, local population and asylum seekers temporarily found each other constructively in Lesvos and fought together against their common enemy, the ‘invisible’ SARS‐CoV‐2 virus to keep everyone alive. In May, the acute COVID‐19 restrictions were gradually relaxed, the different asylum‐related stakeholders returned to their earlier positions, and asylum‐related migrants became again (un)necessary residues of the state, utilised if needed for other purposes. The tensions in Lesvos grew again, as before the pandemic, including physical attacks against asylum‐related migrants and NGOs (see BBC 2020a; Fallon 2020b).

## Conclusions

This paper situated the COVID‐19 pandemic in the broader context of biogeopolitics, using an empirical case from the EU southeastern borderlands between Greece and Turkey. Biogeopolitics was evident in the governance and (mis)management of asylum‐related migrants in the contexts of the COVID‐19 pandemic in 2020. These people were in between states (Turkey/ Greece/ the EU), having left their country of origin and being on their journeys to the country of destination. As this case illustrates, vulnerable people are neglected in times of serious economic, social and political challenges and distress, such as the COVID‐19 pandemic, and they are utilised by the authorities for other purposes. The biopolitical governance and (mis)management of asylum‐related migrants on both sides of the EU southeastern border were connected to the aims and practices of key state stakeholders to develop and accomplish their preferred geopolitical goals in the region. In the biogeopolitics of COVID‐19, such a top‐down approach included dichotomised policies and practices to keep these migrants alive or let them die (also from the pandemic), and using them to threaten geopolitically rival states and organisations. The state might keep these unwanted people alive but does not want these residues of the state to mix up with the citizens. However, the migrants also show bottom‐up agency by organising themselves in the context of the COVID‐19 threat, promoting new political identity by gaining international attention for their case and creating solidarity among themselves – even if they were not fully able to protect themselves from the potential threat of the virus itself. During the initial acute crisis, the different stakeholders joined together to fight against this threatening virus but after they returned back to their positions.

The media discussions and the activities of the states illustrate how COVID‐19 has geopolitical dimensions. The actions against COVID‐19 focused on states, state territories and borders and citizens within those states. These actions related to the SARS‐CoV‐2 virus foster the role of states as the key geopolitical references to imagine, discuss and ultimately act against COVID‐19. This ‘Chinese virus’ (as President of the United States Trump purposefully referred to COVID‐19 a few times) seriously ‘attacked’ Italy, Iran, the United States and practically all countries, facilitated by the mobility of people across national borders. Later, the borders were closed, the countries were locked down and the virus was gradually ‘strangled’ in these territorial traps. A pandemic spreading across the globe and impacting the global economy resuscitated the state as the first and foremost (even if not always so well‐performing) agent to impact the lives of its population. Those people who do not belong (yet or not anymore) to the state are neglected and utilised to foster one’s own state and threaten the states of others. The forthcoming research about the impacts of the COVID‐19 pandemic may also address other very vulnerable people within states, such as undocumented migrants, many of whom are not allowed or are afraid to use necessary medical services provided by the state and other authorities.
